# Supplementary material for: Cremastra appendiculata Polysaccharides Alleviate Neurodegenerative Diseases in Caenorhabditis elegans: Targeting Amyloid-β Toxicity, Tau Toxicity and Oxidative Stress
Source: Int J Mol Sci. 2025 Apr 20;26(8):3900. doi: 10.3390/ijms26083900 (PMC12027758; doi:10.3390/ijms26083900)

Supplementary figure captions

Supplementary Fig. S1. Effects of CAP on the pharyngeal pump and body size of AD model *C. elegans*. The pharyngeal pump of CL4176 (A) and BR5270 (B); The body width of CL4176 (C) and BR5270 (D); The body length of CL4176 (E) and BR5270 (F). Bars with no letters in common are significantly different ($P < 0.05$).

Supplementary tables

Supplementary Table S1

Primer sequence for qRT-PCR

Primer	Primer sequences (5'-3')
act-1	F: AGAAGAGCACCCAGTCCTCC<br>R: GAAGCGTAGAGGGAGAGGAC
sod-3	F: CCAACCAGCGCTGAAATTCAATGG<br>R: GGAACCGAAGTCGCGCTTAATAGT
daf-16	F: CCAGACGGAAGGCTTAAACT<br>R: ATTCGCATGAAACGAGAATG
hsp-70	F: AGCCCGTTGTTGAGGTTGAA<br>R: CCCGTACAGAATGCCCAAGT
gst-4	F: TCCGTCAATTCACTTCTTCCG<br>R: AAGAAATCATCACGGGCTGG
skn-1	F: GAGAGAAGGGCACACGACAA<br>R: TCGAGCATTCTCTTCGGCAG
ctl-1	F: TGTCGTTCATGCCAAGGGAG<br>R: GATCCCGATTCTCCAGCGAC
daf-2	F: CGGTGCGAAGAGAGGATATT<br>R: TACAGAGGTCGCCGTTACTG
hsf-1	F: ATGCAGCCAGGATTGTCGAA<br>R: GCACGTTTTGAGTTGGGTCC

Supplementary Table S2

Effects of CAP on the lifespan of AD model *C. elegans*

	Concentration(mg/mL)	Average lifespan(d)	Maximal lifespan(d)	*P*-value
CL4176	Control	14.41±0.91	25	-
	0.1	18.62±0.98	27	0.004
	0.5	17.02±1.26	27	0.028
	1.0	19.66±1.03	27	<0.001
	1.5	18.60±0.97	29	0.006
	2.0	17.81±1.16	29	0.01
BR5270	Control	12.48±0.34	17	-
	0.1	12.76±0.36	18	0.450
	0.5	13.60±0.33	18	0.019
	1.0	13.83±0.28	19	0.011
	1.5	13.89±0.35	20	0.013
	2.0	14.57±0.44	22	<0.001

Note: $P < 0.05$ indicates a significant difference compared with the control, and $P > 0.05$ indicates no significant difference compared with the control, the same as below.

Supplementary Table S3

Effect of CAP on paralysis in CL4176

Concentration(mg/mL)	Average non-paralysis time (h)	Non-paralysis time(h)	*P*-value
Control	36.67±1.23	48	-
0.1	38.93±1.25	48	0.405
0.5	39.33±1.56	52	0.100
1.0	40.80±1.57	54	0.025
1.5	42.53±1.78	56	0.011
2.0	45.07±2.20	60	0.003

Note: $P < 0.05$ indicates a significant difference compared with the control, and $P > 0.05$ indicates no significant difference compared with the control, the same as below.

Supplementary Table S4

Effects of CAP on the lifespan of AD model *C. elegans* under oxidative stress

	Concentration(mg/mL)	Average lifespan(h)	Maximal lifespan(h)	*P*-value
CL4176	Control	3.60±0.74	10	-
	0.1	6.47±0.99	12	0.017
	0.5	8.13±1.14	14	0.001
	1.0	10.27±0.92	16	<0.0001
	1.5	12.20±0.99	18	<0.0001
	2.0	12.87±1.06	18	<0.0001
BR5270	Control	4.57±0.23	8	-
	1.0	5.53±0.30	9	0.012
	1.5	5.87±0.34	10	0.002
	2.0	6.53±0.38	11	<0.001

Note: $P < 0.05$ indicates a significant difference compared with the control, and $P > 0.05$ indicates no significant difference compared with the control, the same as below.

Supplementary Table S5

Effect of CAP on the lifespan of BR5270 under heat stress

Concentration(mg/mL)	Average lifespan(h)	Maximal lifespan(h)	*P*-value
Control	4.80±0.37	7	-
1.0	5.73±0.38	8	0.070
1.5	6.13±0.42	9	0.016
2.0	6.67±0.36	9	0.002

Note: $P < 0.05$ indicates a significant difference compared with the control, and $P > 0.05$ indicates no significant difference compared with the control, the same as below.

Supplementary Fig. S1

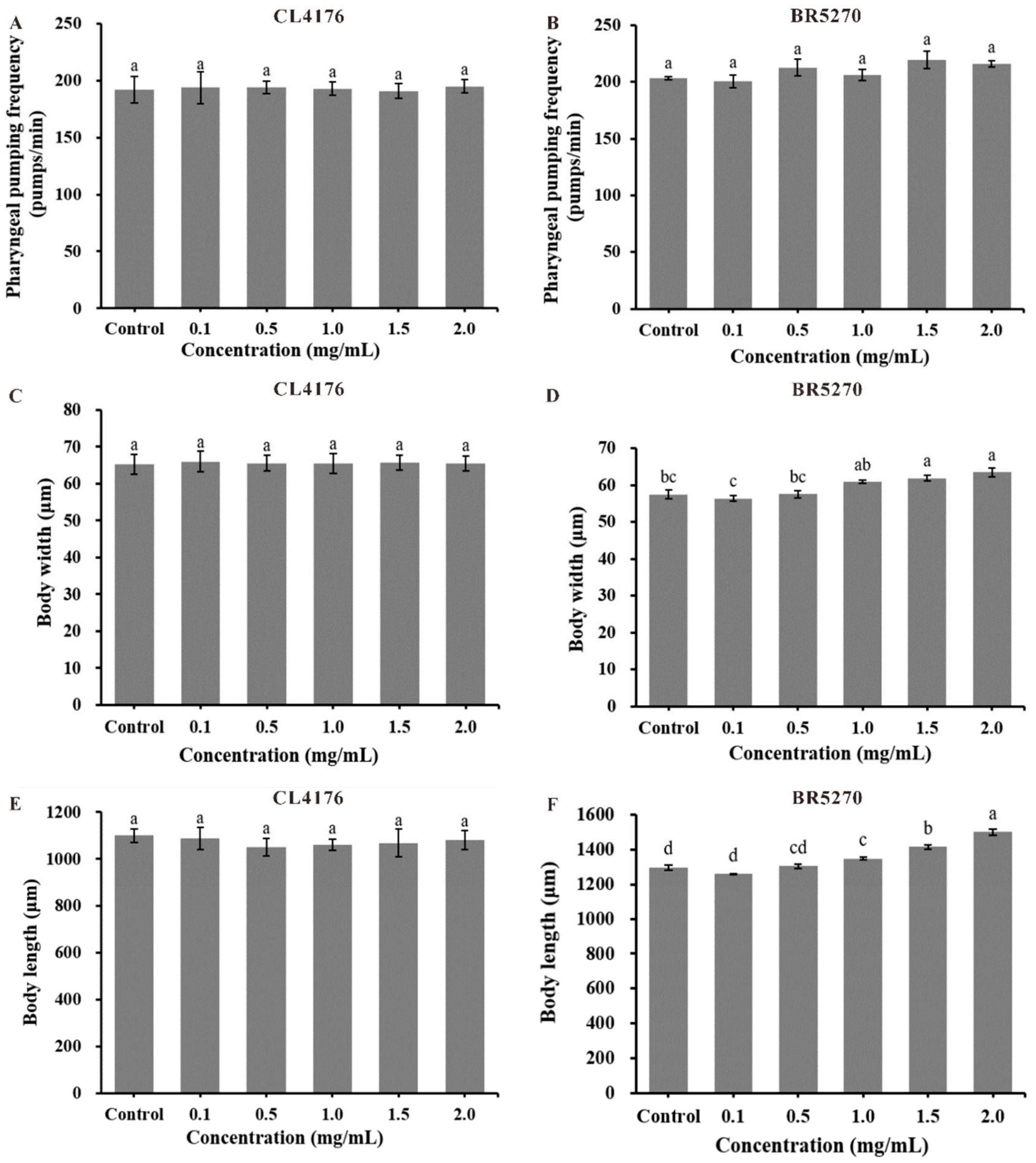

Supplement: Supplementary file 1 [file ijms-26-03900-s001.zip › ijms-3556912-supplementary.pdf]
